# Supplementary material for: Visually evoked responses are enhanced when engaging in a video game
Source: Eur J Neurosci. 2020 Aug 30;52(12):4695–708. doi: 10.1111/ejn.14924 (PMC7818444; doi:10.1111/ejn.14924)
Supplement: Supplementary file 1 — Figure S1‐S6 [file EJN-52-4695-s001.docx]

# Supplementary Information: Visually evoked responses are enhanced when engaging in a video game


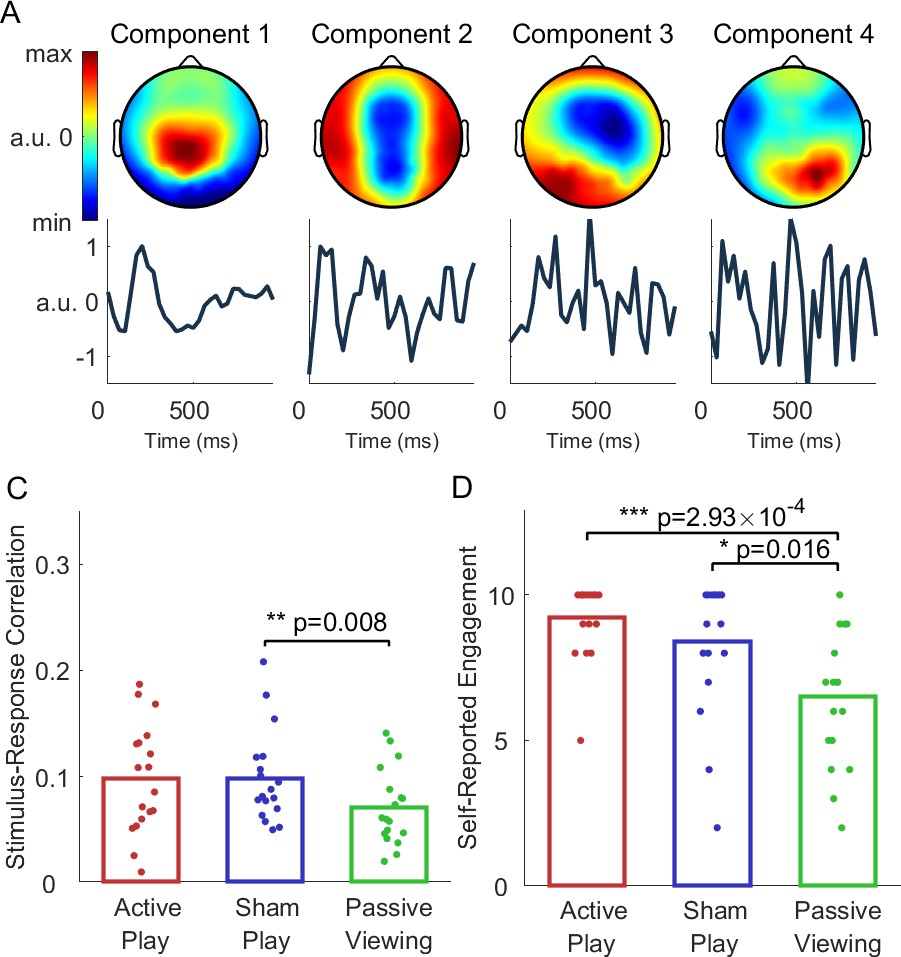


**Fig. S1. Reproducibility of effect with temporal visual contrast.** We tested whether the effect of motor engagement on visual evoked responses would be reproduced when regressing the EEG onto visual contrast, as opposed to the optic flow used in the main analysis; compare with Fig [2.](#_bookmark1) (**A**) (Top row) The spatial response functions of the first four components are largely consistent with those found using optic flow. (Bottom row) The temporal response function of the first component is consistent with that found using optic flow. The remaining components exhibit more high-frequency activity than found in the main analysis. (**B**) Reproducing the effect found with optic flow, sham play yielded significantly higher total SRC compared to passive viewing (*p* = 0*.*008, two-tailed, paired Wilcoxon signed rank test, *n* = 18). Active play also produced higher SRC compared to passive viewing, but the difference fell short of statistical significance (*p* = 0*.*14).


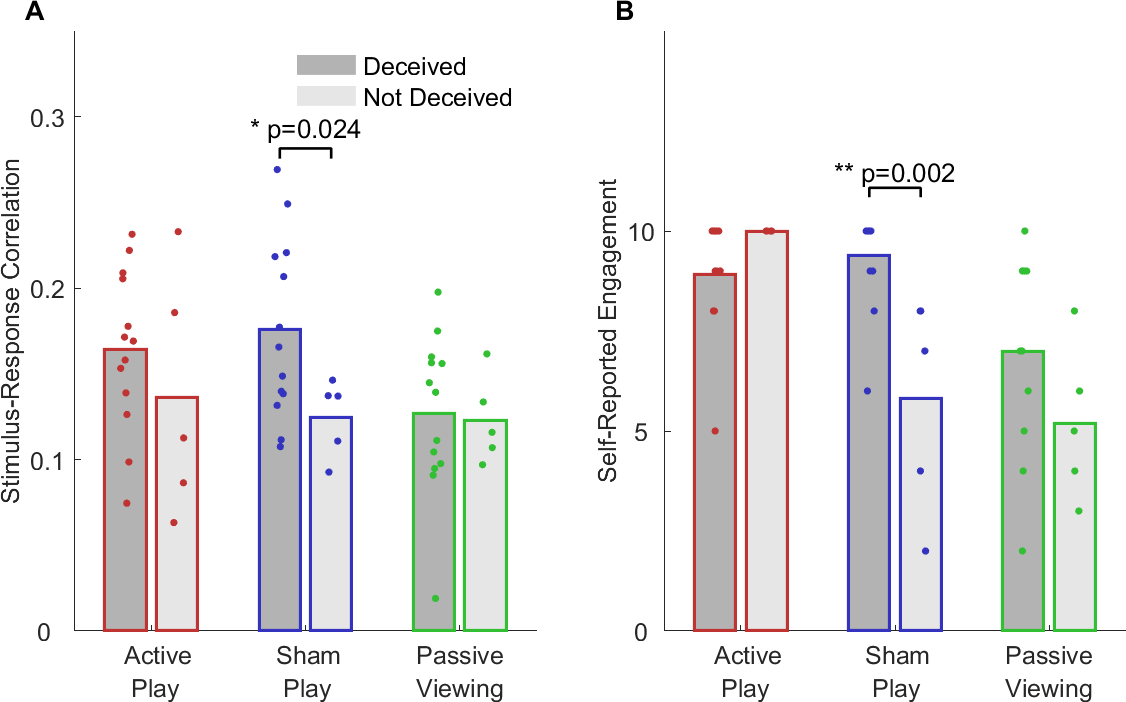


**Fig. S2. Enhanced visual evoked responses in deceived subjects.** (A) Of the 18 study participants, *n* = 13 perceived neural control throughout the sham play trials while *n* = 5 were not deceived. Computing SRC separately within each group, we found enhanced visual evoked responses in the deceived subjects during sham play (*p* = 0*.*024, one-tailed Wilcoxon rank sum test). No significant difference was found between deceived and non-deceived subjects during active play or passive viewing. (B) Deceived subjects reported significantly higher engagement during sham play (*p* = 0*.*0026, one-tailed Wilcoxon rank sum test), but not during active play or passive viewing (both *p >* 0*.*05, one-tailed Wilcoxon rank sum test).


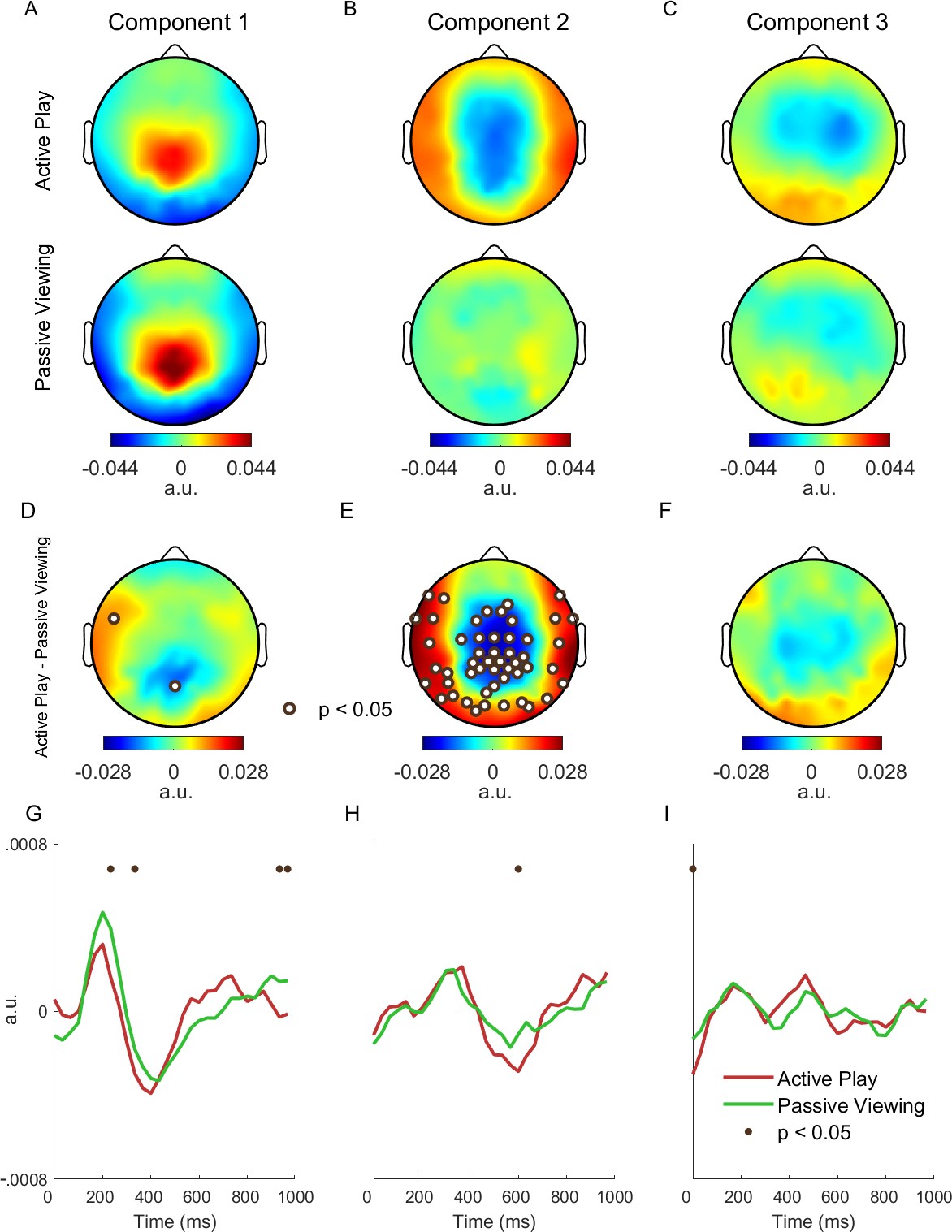


**DRAFT**

**Fig. S3. Spatial and temporal response functions during active play differ from those measured during passive viewing.** (**A**)-(**C**) (Top row) Spatial response functions of the first three components measured during active play. (Bottom row) The spatial response functions measured during passive viewing. (**D**)-(**F**) Significant differences between the spatial response functions of the active play and passive viewing conditions were found over extensive regions of the scalp in Component 2. (**G**)-(**I**) The temporal response functions of the first three components for active play (red) and passive viewing (green). Significant differences were found in all three components, including a stronger response at 600 ms in Component 2.


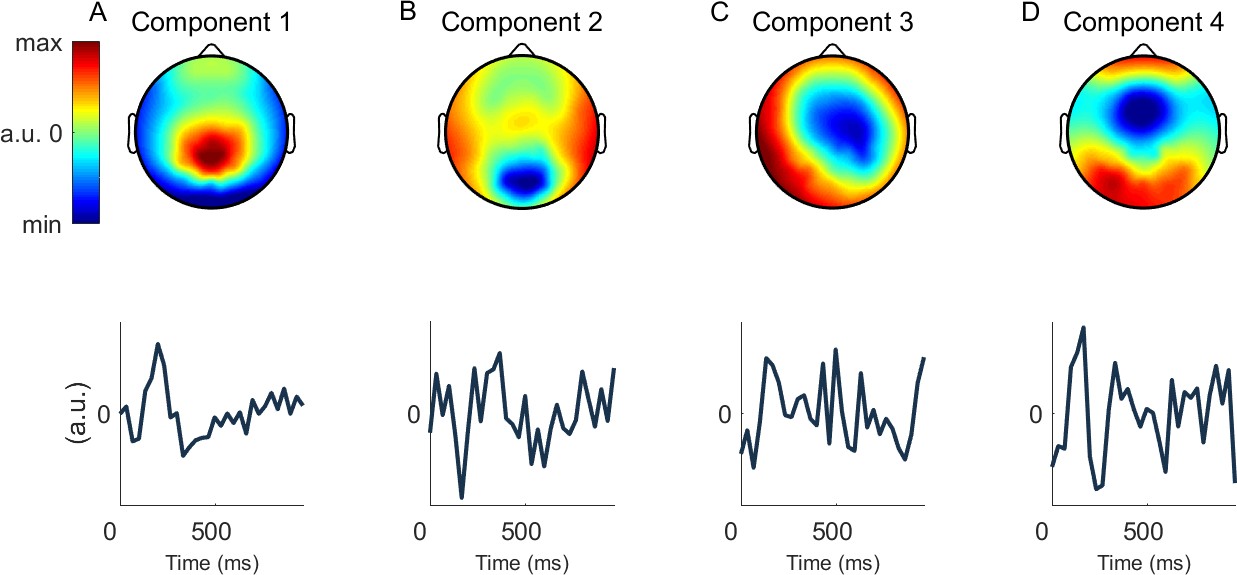


**Fig. S4. Evoked response patterns are reproduced in follow up study.** (**A**)-(**D**) (Top row) Spatial response components of the first four components bear a strong resemblance to those found in the initial study (compare with Fig2A). As in the initial study, the strongest component was expressed over centroparietal electrodes. (Bottom row) The corresponding temporal response functions represent the time course of the evoked response to the optic flow stimulus.


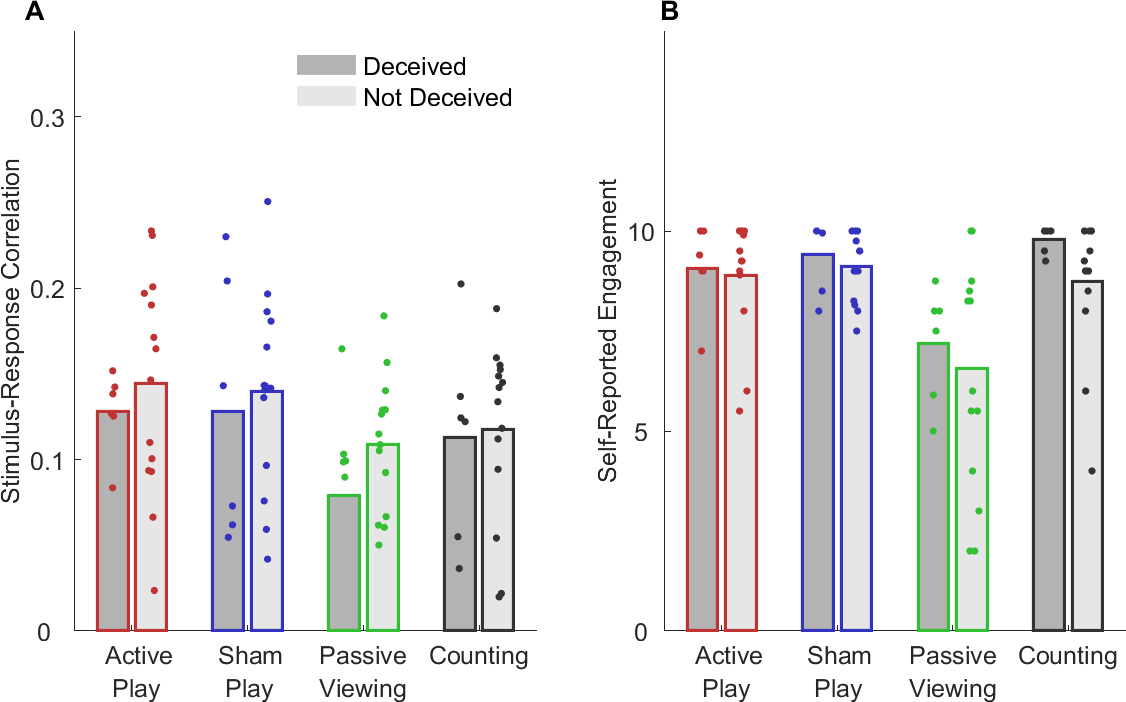


**Fig. S5. No differences in SRC or self-reported engagement between deceived and non-deceived subjects in follow-up study.** Of the 20 follow-up study participants, 6 perceived neural control throughout sham play, while 14 were not deceived. (**A**) Total SRC was not significantly different between deceived and non-deceived subjects for any of the conditions (all *p >* 0*.*05, Wilcoxon rank sum test). (**B**) Similarly, we did not find any significant differences in self-reported engagement between deceived and not deceived subjects (all *p >* 0*.*05).


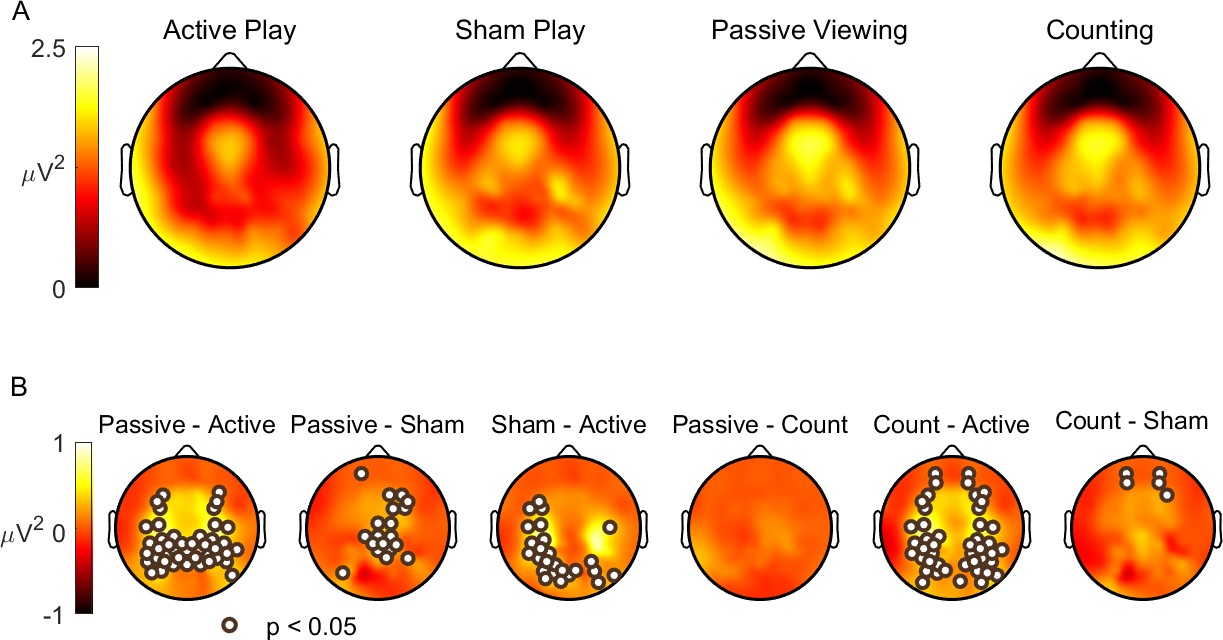


**Fig. S6. No difference in alpha power between counting and passive viewing.** In the initial study, we found a significant decrease in alpha power during sham play relative to passive viewing, suggestive of motor cortex recruitment during sham play. (**A**) We measured alpha power for all four conditions in the follow-up study: active play, sham play, passive viewing, and counting. (**B**) Consistent with the findings of the initial study, we found reduced alpha power over central and parietal electrodes during active and sham play relative to passive viewing (*p <* 0*.*05, *n* = 20, corrected for multiple comparisons by controlling the false discovery rate at 0.05). On the other hand, there were no significant differences in alpha power between passive viewing and the counting task (*p >* 0*.*05, *n* = 20).
